# Long-Term Straw Incorporation under Controlled Irrigation Improves Soil Quality of Paddy Field and Rice Yield in Northeast China

**DOI:** 10.3390/plants13101357

**Published:** 2024-05-14

**Authors:** Peng Zhang, Peng Chen, Tangzhe Nie, Zhongxue Zhang, Tiecheng Li, Changlei Dai, Lili Jiang, Yu Wu, Zhongyi Sun, Shuai Yin

**Affiliations:** 1 School of Water Conservancy and Electric Power, Heilongjiang University, Harbin 150080, China; 2221975@s.hlju.edu.cn (P.Z.);; 2 College of Agricultural Science and Engineering, Hohai University, Nanjing 211100, China; 3 Key Laboratory of Effective Utilization of Agricultural Water Resources, Ministry of Agriculture and Rural Affairs, Northeast Agricultural University, Harbin 150030, China; 4 School of Water Conservancy and Civil Engineering, Northeast Agricultural University, Harbin 150030, China; 5 College of Ecology and Environment, Hainan University, Haikou 570208, China; 6 State Key Laboratory of Remote Sensing Science, Aerospace Information Research Institute, Chinese Academy of Sciences, Beijing 100101, China

**Keywords:** paddy fields, soil quality index, straw incorporation, irrigation regime, soil nutrient management

## Abstract

Soil quality is an indicator of the ability to ensure ecological security and sustainable soil usage. The effects of long-term straw incorporation and different irrigation regimes on the yield and soil quality of paddy fields in cold regions remain unclear. This study established four treatments: controlled irrigation + continuous straw incorporation for 3 years (C3), controlled irrigation + continuous straw incorporation for 7 years (C7), flooded irrigation + continuous straw incorporation for 3 years (F3), and flooded irrigation + continuous straw incorporation for 7 years (F7). Analysis was conducted on the impact of various irrigation regimes and straw incorporation years on the physicochemical characteristics and quality of the soil. The soil quality index (SQI) for rice fields was computed using separate datasets for each treatment. The soil nitrate nitrogen, available phosphorus, soil organic carbon, and soil organic matter contents of the C7 were 93.51%, 5.80%, 8.90%, and 8.26% higher compared to C3, respectively. In addition, the yield of the C7 treatment was 5.18%, 4.89%, and 10.32% higher than those of F3, C3, and F7, respectively. The validity of the minimum data set (MDS) was verified by correlation, *E_f_* and *E_R_*, which indicated that the MDS of all treatments were able to provide a valid evaluation of soil quality. The MDS based SQI of C7 was 11.05%, 11.97%, and 27.71% higher than that of F3, C3, and F7, respectively. Overall, long-term straw incorporation combined with controlled irrigation increases yield and soil quality in paddy fields in cold regions. This study provides a thorough assessment of soil quality concerning irrigation regimes and straw incorporation years to preserve food security and the sustainability of agricultural output. Additionally, it offers a basis for soil quality diagnosis of paddy fields in the Northeast China.

## 1. Introduction

Rice holds immense significance as a staple food for nearly 50% of the global population [1,2]. However, the productivity of rice cultivation has encountered stagnation in many rice-growing regions [3], largely due to the influence of climatic conditions, field management practices [4], and soil quality [5]. Among these factors, soil quality is shaped not only by soil genesis but also by various factors associated with soil utilization and management [6], including straw incorporation and irrigation regimes [7,8]. Enhancing soil quality via field management will promote sustainable agricultural management approaches [9], attain food security, and contribute to the preservation of agro-ecosystems [10].

In recent years, organic amendments such as manure, compost, and straw have been used to improve the environment for rice production [11]. Among them, straw incorporation is recognized as an effective method to maintain or improve rice yield and soil quality [12]. In the short term, straw incorporation offered the advantages of mitigating environmental pollution caused by straw burning and piling [13], while simultaneously improving soil structure and microbial activity [12,14]. The long-term consequences of straw incorporation on the soil quality in paddy fields have not, however, been thoroughly studied. Li et al. [15] concluded that long-term straw incorporation enhanced soil carbon and nitrogen content and facilitated seed yield enhancement by synchronizing nitrogen demand during rice growth. Yang et al. [16] concluded that long-term straw incorporation can increase rice quality. Nonetheless, the benefits of long-term straw incorporation were not universally applicable to paddy fields [17], and the stabilization and augmentation of soil organic carbon did not consistently exhibit a positive or linear correlation with the quantity of straw incorporated into the field [18]. Excessive amounts of straw in croplands may have adverse effects on crop yields due to elevated temperatures and limited oxygen availability [19]. The release of chemicals during the breakdown of straw can impede the growth and development of crops [20].

Furthermore, prolonged flood irrigation combined with straw decomposition in paddy fields often resulted in a rapid decline in soil oxygen levels, leading to a deterioration in the rice root environment [21]. This phenomenon facilitated the accumulation of ammonium nitrogen, also increased the loss of nitrogen during the nitrification-denitrification process [22]. Conversely, water-saving irrigation is currently being widely promoted [23], as it reduced the water consumption in the field [24]. Additionally, water-saving irrigation helped to diminish soil nitrogen loss [25,26]. Some studies have shown that straw incorporation under water-saving irrigation increased soil organic carbon sequestration [27]. The soil microenvironment and straw decomposition process were modified by the implementation of water-efficient irrigation, thereby impacting the productivity of paddy fields. However, the specific relationships between soil physicochemical property and soil quality under different water management and straw incorporation conditions remained unclear. Furthermore, thorough assessments of soil indicators have been lacking in previous research [28,29]. Therefore, a comprehensive evaluation of soil quality considering both straw incorporation and irrigation regimes is imperative for ensuring food security and sustaining agricultural production [30].

Soil quality can be inferred from management-induced changes in soil physicochemical properties [31]. Various methods have been developed for assessing soil quality [32,33]. The most commonly used method for assessment was based on the soil quality index (SQI), as it combined multiple indicators into a composite index using a scoring function [34,35,36], this method effectively handled the multivariate datasets generated by experiments and reflected the trends in soil quality changes [9]. It should be noted that the minimum data set (MDS) approach, as compared to the total data set (TDS), reduced data redundancy and considered the complex interrelationships among multiple indicators [7] and offered the advantages of adaptability and operability [37]. So far, the combined effect of straw incorporation and different irrigation practices on soil quality assessment has been limited [38,39]. Hence, it is imperative to investigate whether MDS based SQI (MDS-SQI) calculation could produce superior outcomes when subjected to varying water management and straw incorporation treatments.

The northeastern region of China holds significant importance as a grain-producing area and has emerged as a prominent rice production base in China [40,41]. However, the soil quality in this region has deteriorated rapidly as a result of long-term irrational farming practices [42]. Additionally, a considerable amount of straw has accumulated over the years of rice cultivation. Consequently, straw incorporation has become an optimal measure to improve soil quality [43,44]. Furthermore, the limited availability of irrigation resources in agriculture has expedited the promotion of local water-saving irrigation techniques [45]. Some studies have pointed out that the combination of water-saving irrigation and nitrogen fertilizer improved rice yield in Northeast China [46]; however, the studies under long-term straw incorporation conditions are not sufficient. Therefore, the objectives of this study were: (1) to analyze the effects of different straw incorporation years and irrigation regimes on soil nutrients, and (2) to screen the indicators of the MDS to calculate the SQI and thus to assess the effects of straw incorporation years and different irrigation regimes on the soil quality of paddy fields. This exploration aims to establish a foundation for future soil quality diagnosis and sustainable development of paddy fields in cold region in Northeast China.

## 2. Results

### 2.1. Soil Physical and Chemical Properties

**Figure 1:**
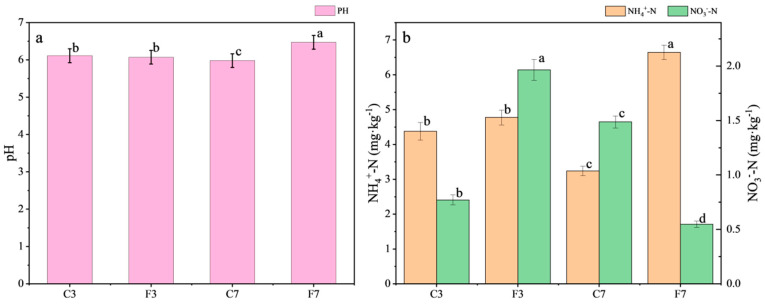

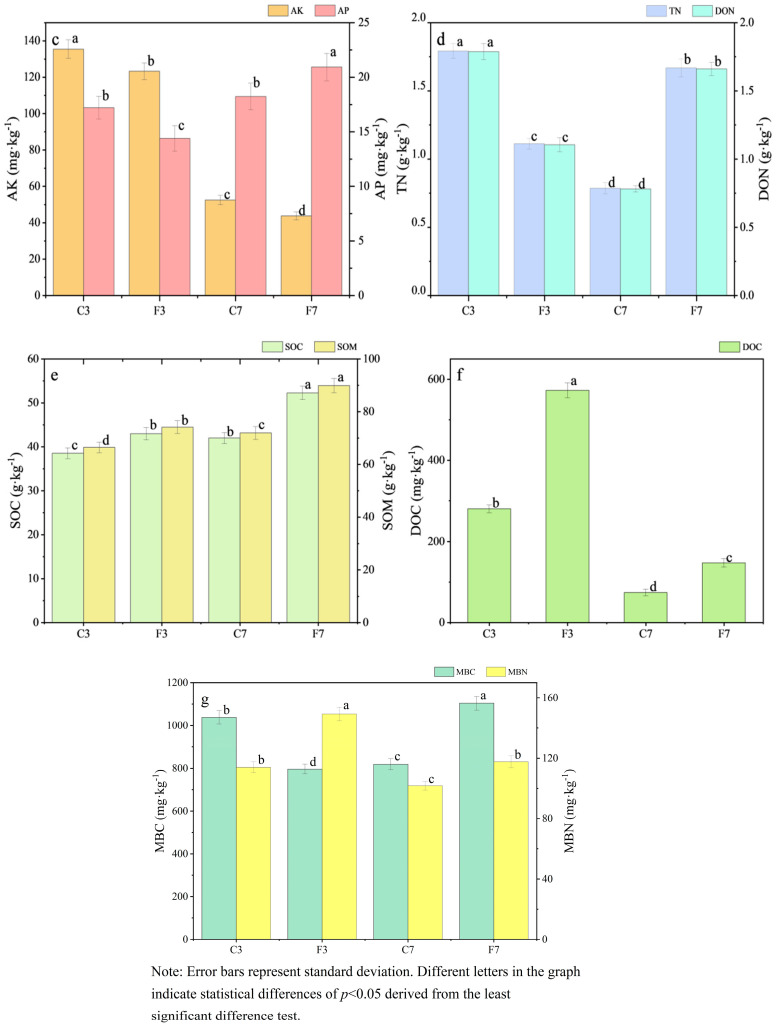
Soil (**a**) pH, (**b**) NH_4_^+^-N and NO_3_^−^-N, (**c**) AK and AP, (**d**) TN and DON, (**e**) SOC and SOM, (**f**) DOC, (**g**) MBC and MBN of paddy fields in different treatments. Abbreviations: pH: Soil pH; NH_4_^+^-N: Ammonium Nitrogen; NO_3_^−^-N: Nitrate Nitrogen; AK: Available Potassium; AP: Available Phosphorus; DOC: Dissolved Organic Carbon; SOC: Soil Organic Carbon; SOM: Soil Organic Matter; TN: Total Nitrogen; DON: Dissolved Organic Nitrogen; MBC: Microbial Carbon; MBN: Microbial Nitrogen.

Compared to the F3, soil pH (pH) (Figure 1a), ammonium nitrogen (NH_4_^+^-N) (Figure 1b), available phosphorus (AP) (Figure 1c), soil organic carbon (SOC) (Figure 1e), soil organic matter (SOM) (Figure 1e), total nitrogen (TN) (Figure 1d), microbial carbon (MBC) (Figure 1g), and dissolved organic nitrogen (DON) (Figure 1d) showed higher values in F7 with increases of 6.41%, 39.79%, 45.28%, 21.75%, 21.32%, 49.11%, 38.61%, and 55.14%, respectively, while nitrate nitrogen (NO_3_^−^-N) (Figure 1b), dissolved organic carbon (DOC) (Figure 1f), available potassium (AK) (Figure 1c), and microbial nitrogen (MBN) (Figure 1g) exhibited significant reductions (*p* < 0.05). NO_3_^−^-N, AP, SOC, and SOM levels in C7 were higher than those in C3 by 93.51%, 5.80%, 8.90%, and 8.26%, respectively. These findings suggested that the straw incorporation years had a positive impact on soil physicochemical properties, with a more pronounced effect observed under flooded irrigation compared to controlled irrigation.

The F3 resulted in lower AK, AP, TN, DON, and MBC compared to the C3, with reductions of 8.91%, 16.46%, 37.81%, 40%, and 23.28%, respectively, despite the same straw incorporation years. Similarly, when straw was incorporated for 7 years, the pH, NH_4_^+^-N, AP, DOC, SOC, SOM, TN, DON, MBC, and MBN of F7 were higher than C7 by 8.01%, 104.94%, 14.69%, 99.31%, 24.65%, 25.02%, 107.39%, 34.93%, and 15.34% respectively (*p* < 0.05). The largest increases were in NH_4_^+^-N, DOC, and TN.

### 2.2. Rice Yields in Different Treatments

**Figure 2:**
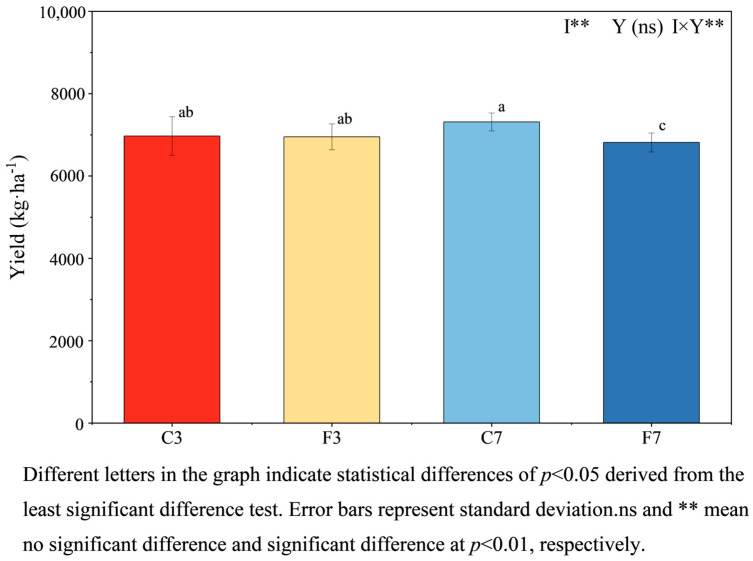
Rice yields in different treatments. Note: I: irrigation regime; Y: year.

Rice yields under different treatments was observed to follow the order of C7 > C3 > F3 > F7 (Figure 2). The C7 treatment showed 4.89%, 5.72%, and 11.27% more than C3, F3, and F7 respectively. In terms of the same straw incorporation years, rice yields were higher in all controlled irrigation treatments compared to flooded irrigation treatments. The rice yield was significantly affected by the irrigation regime, while the straw incorporation year had no significant effect (*p* < 0.05) (Figure 2). However, the combination of straw incorporation years and irrigation regime had a significant effect on yield (*p* < 0.05).

### 2.3. Minimum Data Set Filtering

Soil nutrients were analyzed separately for each treatment by principal component analysis (Figure S1). Taking F3 as an example, PC1, PC2, PC3, and PC4 (with eigenvalues greater than 1) accounted for 33.78%, 31.28%, 20.60%, and 7.60% of the variability, respectively. Cumulatively, they contributed to a total variance of 92.35%. Additionally, the metric variance of each indicator was greater than 80%, indicating that the first four principal components effectively captured the information of each indicator and the overall soil quality (Table 1). From Table 1, the high weight indicators under F3 were PC1: MBC, TN, MBC/MBN, DON; PC2: DOC, SOC, SOM; PC3: NH_4_^+^-N, NO_3_^−^-N; and PC4: C/N. Based on the Tables S1 and S2, the MDS for F3 were identified as MBC/MBN, DOC, NO_3_^−^-N, and C/N. The differences in MDS for evaluating paddy soil quality under different treatments indicated variations in the main limiting factors among the treatments (Table 2).

### 2.4. Soil Quality Index

**Figure 3:**
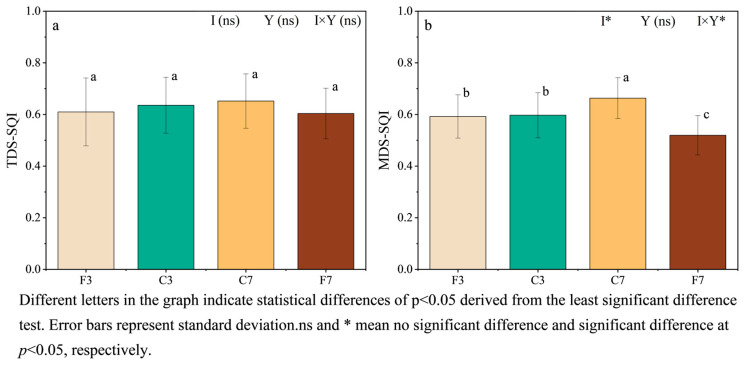
(**a**)TDS-SQI and (**b**)MDS-SQI of paddy soil in different treatments. Note: I: irrigation regime; Y: year.

The weight value occupied by SQI was calculated using Equation (1) (Tables S3 and S4), the SQI for TDS and MDS were calculated separately (Figure 3). To verify the validity of the MDS, ANOVA was performed on the TDS-SQI and MDS-SQI values. The results showed that there was no significant difference in TDS-SQI among treatments. The TDS-SQI values were ranked as follows: C7 > C3 > F3 > F7, with C7 having a higher SQI of 2.58%, 6.94%, and 8.02% compared to C3, F3, and F7, respectively. Additionally, neither the irrigation regime nor the straw incorporation years had a significant effect on the TDS-SQI (*p* < 0.05) (Figure 3a).

The MDS-SQI values were followed the same ranking order: C7 > C3 > F3 > F7, with C7 exhibiting a higher SQI of 11.05%, 11.97%, and 27.71% compared to C3, F3, and F7, respectively. The irrigation regime had a significant effect on MDS-SQI values of paddy soils, while the straw incorporation years did not have a significant effect on MDS-SQI. However, when the two factors were interacted, the MDS-SQI showed a significant effect (*p* < 0.05) (Figure 3b).

The TDS-SQI and MDS-SQI were significantly correlated for each treatment (*p* < 0.01). Furthermore, the *E_f_* and *E_R_* were calculated using Equations (3) and (4) to validate the rationality, respectively. The *E_f_* values measured for F3, C3, C7, and F7 were 0.67, 0.63, 0.78, and 0.72, respectively, and the ER values were 0.11, 0.16, 0.08, and 0.01. These findings suggested that the MDS was a more effective evaluation method for soil quality compared to the TDS.

### 2.5. Correlation between Rice Yield and MDS-SQI

**Figure 4:**
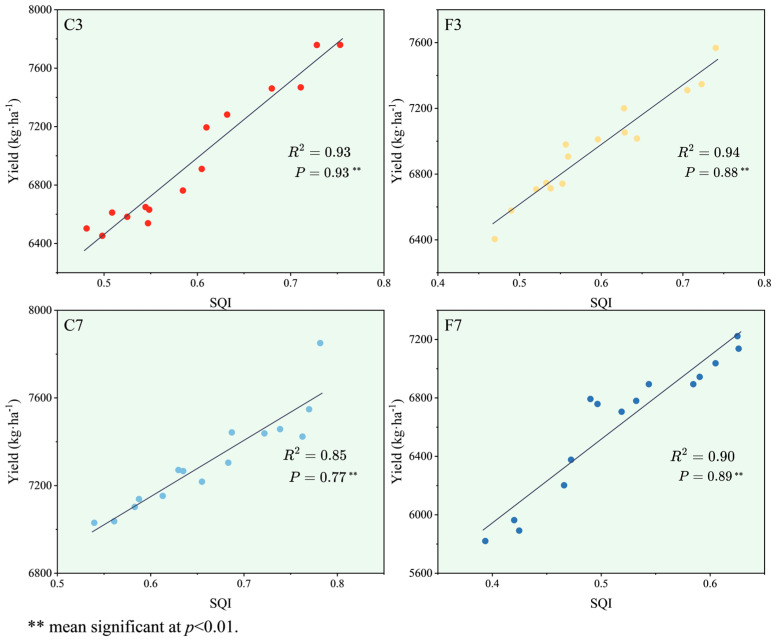
Correlation analysis of rice yield and SQI under different treatments. Note: *P* is the correlation between SQI and yield.

As the MDS-SQI increased, the yields of rice for the F3 (R^2^ = 0.93), C3 (R^2^ = 0.94), C7 (R^2^ = 0.85), and F7 (R^2^ = 0.90) treatments also increased (Figure 4). The rice yield in the F3, C3, C7, and F7 were significantly and positively correlated with their corresponding MDS-SQIs (*p* < 0.01), indicating that MDS-SQI correctly evaluates the soil quality.

## 3. Discussion

### 3.1. Changes in Soil Physicochemical Properties

In addition to being a complete indication of soil chemical characteristics, soil pH is essential for controlling microbial activity and nutrient availability. He et al. [47] concluded that controlling soil water condition increased soil pH, which was inconsistent with the conclusions of this study. Our findings revealed a significant decrease in pH after 7 years of continuous straw incorporation under controlled irrigation. The anaerobic decomposition of straw incorporated, which generated reducible organic acids, might be responsible for this reduction in pH [48]. Conversely, we observed the highest pH in the F7 treatment. This was because persistent flooding conditions led to a lowering of redox potential, which promoted pH elevation [49]. The availability of NH_4_^+^-N/NO_3_^−^-N directly affected crop nutrient uptake [50]. Our study revealed that under flooded conditions, NO_3_^−^-N/NH_4_^+^-N decreased with increasing years of straw incorporation. In contrast, under controlled irrigation conditions, we observed the opposite trend. Previous studies have demonstrated the significant impact of soil acidification on NO_3_^−^-N/NH_4_^+^-N [51,52]. The increase in pH might restrict the movement of NO_3_^−^-N through the root system [53], thereby affecting nitrogen metabolism. Moreover, anaerobic environments favored the accumulation of NH_4_^+^-N [49].

According to previous studies, incorporating straw into the field might increase the potassium content of the soil and reduce the demand for potash fertilizer [54]. However, as the straw incorporation years increased, our study revealed a considerable decline in AK content. This was especially true for the C7 and F7 treatments, where AK content dropped below the crucial threshold of 100 mg kg^−1^, suggesting a serious deficiency. The long-term application of nitrogen, which enhanced crop yields and increased plant uptake of potassium, was probably responsible for the AK reduction. Conversely, long-term straw incorporation might increase the potassium content in the farmland moisture, making it susceptible to runoff losses [54]. The AP content in all treatments exceeded the critical level of 10 mg kg^−1^. The increase in soil pH under water-saving irrigation could reduce the adsorption and immobilization of AP. Conversely, continuous flooded irrigation combined with straw incorporation increased the total soil reductant content, potentially inhibiting AP uptake by rice [48]. Furthermore, a decrease in pH altered the form and availability of elements in the soil solution, leading to phosphorus deficiency [49].

SOM is an essential indication for assessing the level of soil fertility because it provides plants with nutrients and energy for soil microbial activities [55,56]. A previous study indicated a positive relationship between SOM and the productivity of paddy yield [57], and a higher SOM content was associated with the soil’s ability to retain fertilizers and increase yields [58], which is different from this study. The SOM of the F7 was significantly higher than that of the F3, indicating that long-term straw incorporation effectively increased the SOC content, thereby providing more nutrients for plants [59]. Enhanced SOC content could be linked to reduced pH, which inhibited SOC decomposition under controlled irrigation [60]. The DOC content generally constituted less than 2% of the total SOC [61]. This study observed a decrease in DOC content with increasing years of straw incorporation. Long-term tillage practices led to a significant depletion of DOC content, and consecutive years of straw incorporation were insufficient to compensate for this reduction [62].

Soil TN is a key factor in determining soil fertility [63]. Loss of nitrogen from paddy fields usually resulted in nitrogen deficiency [64]. We observed that increasing of incorporating straw years under flooded irrigation significantly enhanced the nitrogen content in the soil. This effect was likely due to the additional nitrogen contributed to the soil through the decomposition of straw, resulting in the formation of humus and other organic compounds. Organic nitrogen is easily stored in soil [65]. The C7 exhibited the highest C/N ratio and significantly higher crop yield (*p* < 0.05). This improvement might be attributed to the involvement of microorganisms in converting straw nitrogen during the process of straw carbon conversion, thereby influencing soil nitrogen [66].

Numerous studies have shown that the amounts of MBC and MBN represent the soil’s ability to store and recycle vital nutrients, making them significant markers of soil microbial fertility [67]. Increasing SOM did not result in a corresponding increase in MBN content in this study. This observation may be attributed to long-term tillage practices that lead to soil disturbance and suppress microbial activity [68]. Furthermore, changes in soil pH due to straw incorporation could also exert an influence on soil microbial biomass [69]. MBC/MBN can effectively characterize the proportion of active carbon to nitrogen in the soil. Some studies have pointed out that straw incorporation replenished carbon and nitrogen organisms in the soil and enriched microbial biomass [70]. Our findings indicated that MBC/MBN differed among treatments, with the highest MBC/MBN observed in F7, followed by C3, C7, and F3. Factors such as nitrogen and phosphorus losses and elevated soil temperature also contributed to a decrease in microbial population, thereby influencing the MBC/MBN.

### 3.2. MDS-Based Soil Quality Evaluation

The TDS and MDS have been widely used in soil quality evaluation [9,71]. The TDS approach considers all identified variables [36], but it has limitations related to indicator redundancy and the complexity of data interpretation [7]. In our study, separate datasets were established for different treatments, and the results revealed no significant difference in TDS-SQI. This suggested that TDS might not be suitable for analyzing changes in soil quality of paddy fields in cold regions. In contrast, the MDS can accurately reflect soil quality, reduces data redundancy, and is highly adaptable [37,72]. The reliability of MDS was verified through correlation, *E_f_* and *E_R_*, indicating that MDS outperformed TDS in effectively and accurately evaluating soil quality, and provided valuable information for farmland management.

Previous studies have demonstrated the positive impact of straw incorporation on soil quality and subsequent crop yield improvement [73]. In the present study, the MDS-SQI for the four treatments followed the order of C7 > C3 > F3 > F7. Furthermore, significant and positive correlations were observed between the MDS-SQI and yields of all treatments (*p*< 0.01), indicating the influence of soil quality on paddy yield across all treatments. Specifically, the SQI values decreased under the flooded irrigation with long-term straw incorporation, whereas SQI values significantly increased (*p*< 0.05) under controlled irrigation with increasing years of straw incorporation. Importantly, the rice yields followed the order of C7 > C3 > F3 > F7, with the SQI level directly determining the yield [74]. Zhu et al. [75] observed that continuous straw incorporation combined with the promotion of soil material cycling enhanced soil fertility and increased crop yields. While initial straw incorporation under flooded irrigation improved soil quality and subsequently increased yields [76], long-term straw incorporation was less beneficial than controlled irrigation in enhancing paddy yield and soil quality. This may be attributed to prolonged flooding negatively affecting rice root growth, with root systems primarily concentrating on the soil surface and inhibiting proper tillering [26]. Additionally, prolonged flooding might accumulate toxic substances in soil, cause a decrease in microbial populations [77], and diminish soil fertility, resulting in lower yields.

This study describes changes in soil nutrient characteristics. However, future studies should also consider soil microbial indicators. Soil microorganisms facilitate nutrient transformation and maintain soil ecological health [78]. Additionally, soil indicators closely related to water management, such as redox potential and soil enzyme activity, should also be included.

## 4. Materials and Methods

### 4.1. Study Area

**Figure 5:**
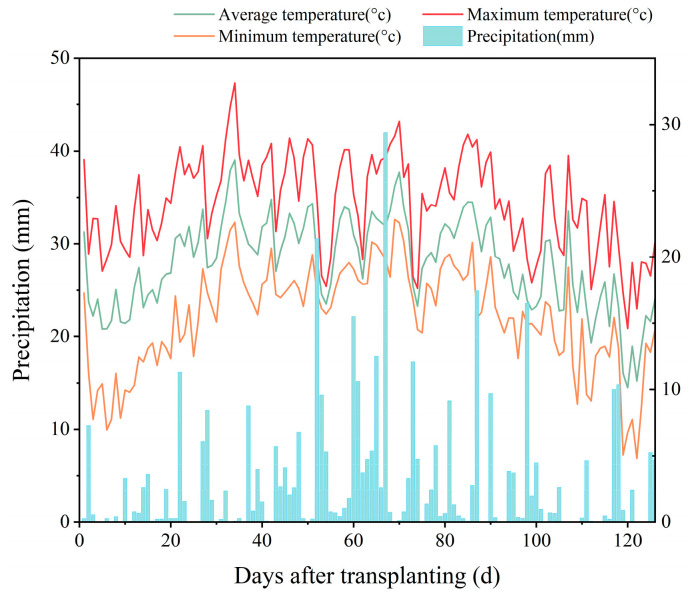
Air temperature and precipitation during the rice growth period in 2023.

The experiment was conducted at Qing’an National Key Station of Irrigation Experiment in Heilongjiang Province (127°40′44′ E, 46°57′29′ N). This station has a cold-temperate continental monsoon climate with low precipitation in spring, which is prone to drought, and early cold spells in autumn. From the rice re-greening stage to maturity, the average precipitation over the years is 550 mm. The soil used for the experiment was sandy clay loam, with a soil tillage thickness of 11.5 cm. Rice has been a major food crop in the study site for over forty years. To increase yields and save water, measures such as water-saving irrigation have been proposed since 2004. Figure 5 shows the maximum, minimum, and average air temperatures together with the amount of precipitation at the experimental site in 2023 during the rice growth period.

### 4.2. Experimental Design

The experimental plots underwent consecutive years of straw incorporation experiments, initiated in 2016 and 2020, respectively, and have been ongoing since then. The basic properties of straw are shown in Table 3. The experiment of this study took place from May to September 2023, encompassing 7 and 3 consecutive years of straw incorporation, respectively. Two irrigation regimes—controlled irrigation (C) and flooded irrigation (F)—were used in this study. Table 4 detailed the experimental treatments and water management during the rice growth period of each treatment; there were a total of four treatments: C3, F3, C7, and F7; three randomized replications were set up for each experimental treatment. The primary local cultivar used throughout the entire experimental cycle was “Suijing18”, planted at a density of 24 cm × 16 cm, with three plants per hill. The fertilization management and straw incorporation standards adopted were in line with the recommendations of Nie et al. [79].

### 4.3. Sample Collection and Determination

At the mature stage of rice, to avoid marginal effects, a buffer of two rows and two columns around the plots was excluded. Yield measurements were taken from rice plants of 3 m^2^ collected separately from each plot and manually threshed [79].

On 23 September 2023, soil samples were collected from the middle of each plot by soil auger at 5 points in the 0~20 cm soil layer and preserved according to the protocol of Zhang et al. [56]. During the soil natural air-drying process, once the soil became crushable, larger sample pieces were broken to expedite drying. The samples were regularly turned to eliminate any debris or stones and were sieved and mixed for soil physicochemical property analyses. The determination of soluble organic nitrogen (DON) could not be performed directly and therefore relied on the differential subtraction method. Table 5 shows the remaining soil physicochemical analysis methods.

### 4.4. Establishment of TDS and MDS Metrics

The TDS considered all the identified variables (Table 5) and the MDS is developed by screening using principal component analysis (PCA) to evaluate the indicators of the quality of the tillage layer. First, the selected indicators were subjected to PCA. The magnitude of the eigenvalues indicated the extent to which the principal components represented the variability in the data. Within each group of principal components, the factor loading variables that contributed most were identified, with high factor loading indicating greater importance within that particular principal component. An indicator with a factor loading close to or reaching 90% of the maximum factor loading in that principal component was considered a high loading indicator and included in the MDS. It is important to determine their interrelationships when a principal component has several indicators with high factor loadings.

The formula for calculating the weight (*W_Ni_*) of each indicator is as follows:

(1) $$W_{Ni}=W_{i}/\sum_{i=1}^{n}W_{i}$$

where: *W_i_* is the variance contribution of all indicators in the dataset, *n* is the number of indicators considered.

### 4.5. Soil Quality Evaluation

The main affiliation scoring functions were categorized into three types: positive S, inverse S, and parabolic. For indicators without well-defined boundaries, a simple linear scoring method [81,82] was employed, where the highest value of the measured value was scored as 1, and the ratio of the other measured values to this highest value was equal to the respective score.

The SQI was calculated using the following formula:

(2) $${SQI}_{i}=\sum_{i}^{n}W_{Ni}\times S_{i}$$

where: *S_i_* denotes the indicator score.

**Figure 6:**
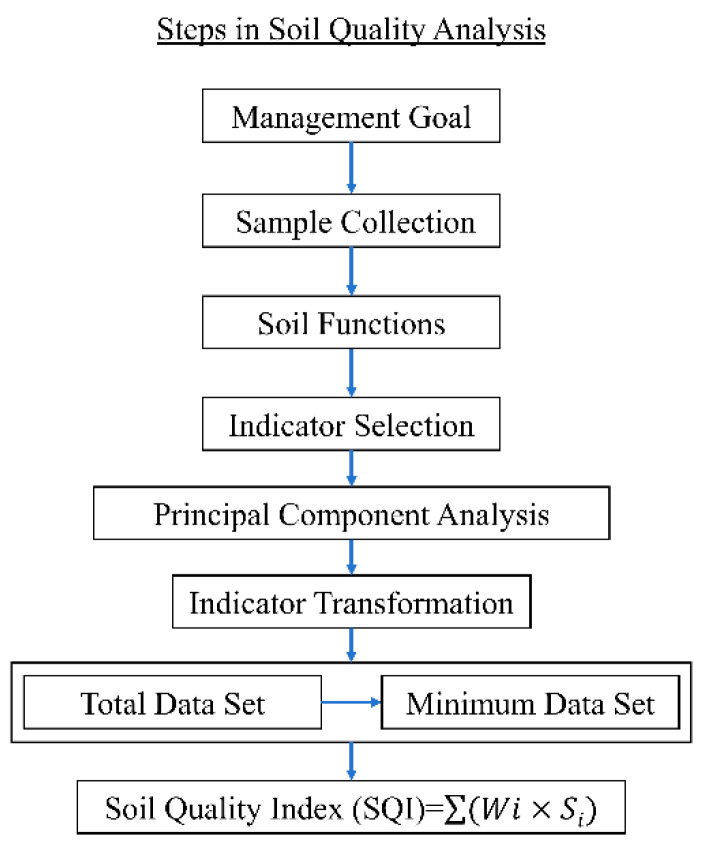
Comprehensive procedures for evaluating and analyzing soil quality.

Finally, the accuracy of SQI was verified using *E_f_* and *E_R_*. The closer *E_f_* is to 1, the more accurate the result, as it indicates that the MDS-SQI is closer to the baseline value. The closer *E_R_* is to 0, the more accurate the result, as it indicates that the MDS-SQI has less deviation from the benchmark value. The calculation formula is as follows [83]:

(3) $$E_{f}=1-\frac{\sum {\left(R_{0}-R_{cal}\right)}^{2}}{\sum {\left(R_{0}-{\bar{R}}_{0}\right)}^{2}}$$

(4) $$E_{R}=\frac{\left|\sum_{i=1}^{n}R_{0i}-\sum_{i=1}^{n}R_{cali}\right|}{\sum_{i=1}^{n}R_{0i}}$$

where: *R_0_* and ${\bar{R}}_{0}$ are the SQI and the average value of the SQI calculated based on the TDS, respectively. *R_cal_* is the MDS-SQI. Figure 6 illustrates the specific procedures for evaluating and analyzing soil quality (refer to Karaca et al. [84] for plotting).

### 4.6. Statistical Analysis

This study employed one-way analysis of variance (ANOVA) to determine the significance of differences in soil physicochemical indicators and yield among different treatments. The interactions between straw incorporation years and irrigation regimes were examined using a two-way ANOVA. The relationship between SQI and yield was assessed by linear regression. Statistical analyses were conducted using SPSS 26.0 (IBM Co., New York, NY, USA), and resulting plots were generated using Origin 2023 (Origin Lab Corporation, Northampton, MA, USA).

## 5. Conclusions

This study presents an analysis of the soil physicochemical quality conditions of rice paddies in cold region, focusing on how successive years of straw incorporation and different irrigation regimes affect soil quality. The results indicated that the C3 showed improvements in AK, AP, TN, DON, and MBC compared to the F3, with increases of 8.91%, 16.46%, 37.81%, 40%, and 23.28%, respectively. The levels of NO_3_^−^-N, AP, SOC, and SOM were higher in the C7 compared to the C3, with increases of 93.51%, 5.80%, 8.90%, and 8.26% respectively. The MDS-SQI values followed the same ranking order: C7>C3>F3>F7, with C7 exhibiting a higher SQI of 11.05%, 11.97%, and 27.71% compared to C3, F3, and F7, respectively. In conclusion, this study demonstrated that long-term straw incorporation under controlled irrigation enhanced rice yield and improved soil quality, while long-term straw incorporation under flooded irrigation did not necessarily result in increased yield and fertilizer conservation. These results help diagnose the condition of the soil quality in cold regions and provide a basis for agricultural practices in rice production in Northeast China, which is important for preserving food security and guaranteeing the sustainability of agricultural output.

## Figures and Tables

**Table 1:** Principal component analysis of soil quality indicators in paddy fields under different treatments.

	F3 Principal Component				C3 Principal Component				C7 Principal Component				F7 Principal Component			
	1	2	3	4	1	2	3	4	1	2	3	4	1	2	3	4
Eigenvalues	4.73	4.38	2.88	1.06	5.12	4.01	2.66	1.82	4.12	3.55	3.38	2.54	3.96	3.76	3.31	2.69
Contribution rate (%)	33.78	31.28	20.60	7.60	36.56	28.67	18.97	12.98	29.42	25.39	24.11	18.13	28.30	26.82	23.66	19.24
Cumulative contribution rate (%)	33.78	65.06	85.66	93.26	36.56	65.23	84.20	97.18	29.42	54.81	79.92	97.05	28.30	55.13	78.79	98.03
Soil indicators																
pH	0.50	0.36	0.70	0.01	0.47	0.22	0.29	0.77	0.40	0.11	0.23	0.87	0.37	0.26	0.04	0.88
NH_4_^+^-N	−0.08	0.14	0.97	−0.07	0.50	0.20	0.74	−0.07	0.36	0.59	0.29	0.59	0.39	0.28	0.12	0.86
NO_3_^−^-N	−0.03	0.02	0.97	0.09	0.04	0.03	−0.35	0.92	0.32	−0.03	0.11	0.93	0.21	−0.02	0.62	0.73
AK	0.27	0.72	−0.13	0.21	0.10	0.52	0.80	0.10	0.06	−0.22	0.89	0.27	0.01	0.95	0.02	0.21
AP	−0.43	0.76	0.43	0.09	0.23	−0.07	0.93	−0.17	−0.01	0.97	0.01	0.10	0.01	0.96	−0.14	0.17
DOC	0.12	0.97	0.15	−0.06	0.17	0.98	0.02	0.12	0.16	0.98	−0.02	−0.05	0.29	−0.03	0.94	0.09
SOC	0.19	0.95	0.13	−0.10	0.24	0.96	0.14	0.06	0.27	0.16	0.92	0.11	0.31	0.46	0.81	0.13

Abbreviations refer to Figure 1.

**Table 2:** Minimum data set in different treatments.

Treatments	MDS
F3	MBC/MBN, DOC, NO_3_^−_^N, C/N
C3	MBC/MBN, SOC, AP, NO_3_^−_^N
C7	DON, SOM, DOC, NO_3_^−_^N
F7	MBN, AK, DOC, pH

Abbreviations refer to Figure 1.

**Table 3:** The basic properties of straw.

Indicators	Values
pH	7.05
Total C (%)	37.06
Total N (g kg^−1^)	6.34
Total P (g kg^−1^)	2.31
Total K (g kg^−1^)	10.67

**Table 4:** Experimental treatments and water management of rice at each growth stage under different irrigation regimes.

Treatments	Irrigation Regimes	Straw Incorporation Years	Regreening Stage	Tillering Stage	Later Tillering	Booting Stage	Flowering Stage	Milk Stage	Mature Stage
C3	Controlled irrigation	3	0~30 mm	70%~100% *θ*_s_	Drainage	80%~100% *θ*_s_	80%~100% *θ*_s_	70%~100% *θ*_s_	Naturally drying
C7									
F3	Flooded irrigation	7	0~30 mm	20~50 mm	Drainage	60~80 mm	60~80 mm	0~30 mm	
F7									

Note: *θ*_s_ refers to soil saturated water content of root layer.

**Table 5:** Methods for soil physicochemical properties analysis.

Soil Indicators	Methods	Abbreviations	References
Soil Acidity and Alkalinity	pH meter	pH	Ran et al. [80]
Ammonium Nitrogen	Indophenol blue colorimetric	NH_4_^+^-N	Nie et al. [79]
Nitrate Nitrogen	UV dual-wavelength	NO_3_^−^-N	
Available Phosphorus	NaHCO_3_-leaching—molybdenum antimony colorimetric	AP	Yang et al. [37]
Available Potassium	Flame photometer analysis	AK	
Total Nitrogen	Kjeldahl method	TN	Qi et al. [81]
Soil Organic Carbon	Dichromate oxidation	SOC	Das et al. [67]
Soil Organic Matter		SOM	
Dissolved Organic Carbon		DOC	
Microbial Biomass Carbon	Chloroform-fumigation technique	MBC	Marion et al. [7]
Microbial Biomass Nitrogen		MBN	

## Data Availability

Data that support the findings of this study are available from the corresponding author upon reasonable request.

## Supplementary Materials

The following supporting information can be downloaded at: https://www.mdpi.com/article/10.3390/plants13101357/s1, Figure S1. Principal component analysis for (a) F3, (b) C3, (c) C7 and (d) F7. Table S1. Correlation coefficients and sum of correlation coefficients of high weight indicators under different treatments. Table S2. Correlation of high factor load indicators under different treatments. Table S3. Weights of TDS evaluation indicators of paddy soil under different treatments. Table S4. Weights of MDS evaluation indicators of paddy soil under different treatments.
