# Supplementary material for: Long-Term Straw Incorporation under Controlled Irrigation Improves Soil Quality of Paddy Field and Rice Yield in Northeast China
Source: Plants (Basel). 2024 May 14;13(10):1357. doi: 10.3390/plants13101357 (PMC11124830; doi:10.3390/plants13101357)
Supplement: Supplementary file 1 [file plants-13-01357-s001.zip › plants-3015801-supplementary.pdf]

Supplementary Materials

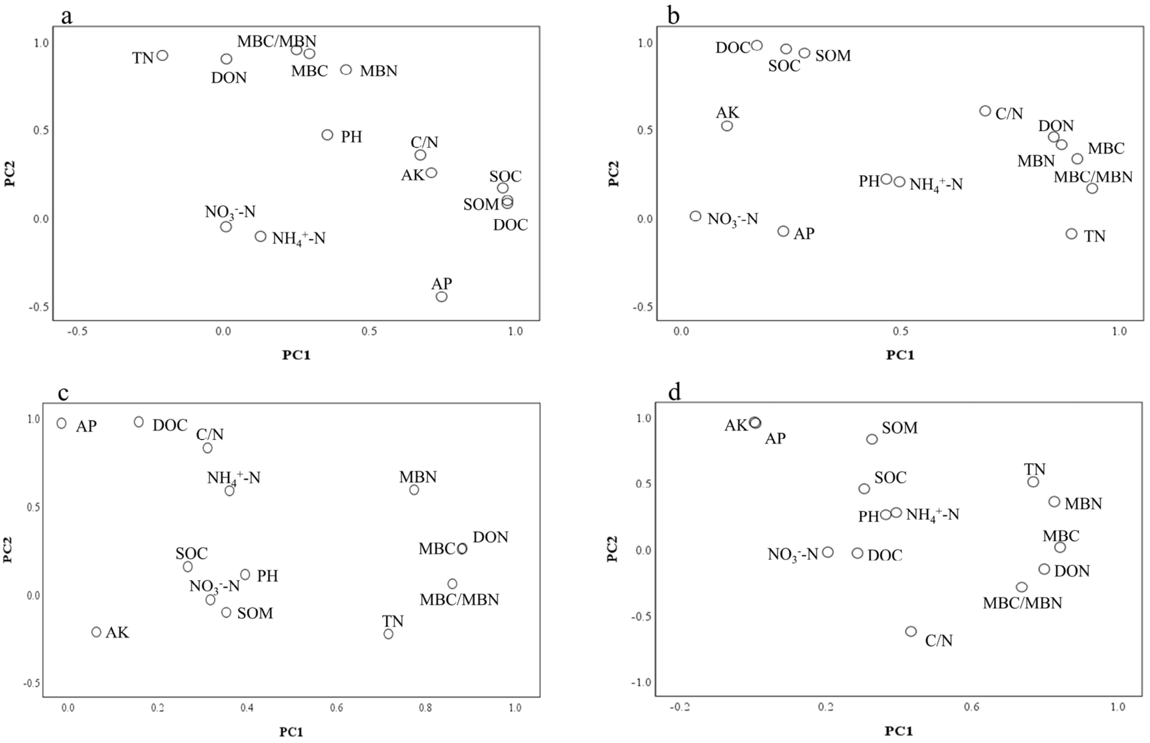

Figure S1. Principal component analysis for (**a**) F3, (**b**) C3, (**c**) C7 and (**d**) F7. Abbreviations refer to Figure 1. C/N: SOC/TN.

Table S1. Correlation coefficients and sum of correlation coefficients of high weight indicators under different treatments.

	**F3**					**C3**					**C7**				**F7**			
**PC1**	**MBC**	**TN**	**MBC/ MBN**	**DON**		**MBC**	**TN**	**MBN**	**MBC/ MBN**		**MBC**	**MBC/ MBN**	**DON**		**MBC**	**TN**	**MBN**	**DON**
Correlation coefficient																		
MBC	1.00	0.74	1.00	0.80	MBC	1.00	0.83	0.99	0.97	MBC	1.00	0.97	1.00	MBC	1.00	0.81	0.91	0.98
TN	0.74	1.00	0.79	0.82	TN	0.83	1.00	0.76	0.93	MBC/ MBN	0.97	1.00	0.97	TN	0.81	1.00	0.98	0.68
MBC/MBN	1.00	0.79	1.00	0.82	MBN	0.99	0.76	1.00	0.94	DON	1.00	0.97	1.00	MBN	0.91	0.98	1.00	0.81
DON	0.80	0.82	0.82	1.00	MBC/ MBN	0.97	0.93	0.94	1.00					DON	0.98	0.68	0.81	1.00
Sum of correlation coefficients	3.54	3.35	3.60	3.43		3.79	3.52	3.69	3.85		2.97	2.94	2.97		3.70	3.46	3.70	3.47
PC2	DOC	SOC	SOM			DOC	DOM	SOC	SOM		SOC	SOM	AK					

Correlation coefficient												
DOC	1.00	0.99	1.00	DOC	1.00	0.98	0.99	1.00	SOC	1.00	0.95	0.80
SOC	0.99	1.00	0.99	DOM	0.98	1.00	1.00	1.00	SOM	0.95	1.00	0.87
SOM	1.00	0.99	1.00	SOC	0.99	1.00	1.00	1.00	AK	0.80	0.87	1.00
				SOM	0.97	1.00	1.00	1.00				
Sum of correlation coefficients	2.99	2.98	2.99		3.94	3.98	3.98	3.96		2.74	2.82	2.66

Abbreviations refer to Figure 1.

Table S2. Correlation of high factor load indicators under different treatments.

Treatments	F3 PC3		C7 PC3		C7 PC4		F7 PC2		F7 PC4	
		NO_3^--N		AP		pH		AK		pH
	NH_4^+-N	0.93**	DOC	0.94**	NO_3^--N	0.80**	AP	0.99**	NH_4^+-N	0.71**

Note: C3 treatments PC3 and PC4 both have only one high factor loading indicator and therefore were placed directly into the MDS. Abbreviations refer to Figure 1.

The PC1 high-weight metric correlations (Table S1) showed that SOM under the F3 treatment had the largest sum of correlation coefficients and was therefore selected into MDS. The high weight indicator MBC/MBN in PC2 had the largest correlation coefficient sum and was therefore selected into the MDS. In PC3, the correlation coefficients of NH_4^+-N and NO_3^--N were r>0.60 (Table S2), and NO_3^--N was selected into the MDS because it had a high factor loading. PC4 had only one high weight indicator C/N, so C/N was selected into the MDS. As a result, the MDS indicators for soil quality evaluation under F3 treatment were SOM, MBC/MBN, NO_3^--N and C/N

Table S3. Weights of TDS evaluation indicators of paddy soil under different treatments.

Soil indicators	F3 Common factor variance	F3 Weight value	C3 Common factor variance	C3 Weight value	C7 Common factor variance	C7 Weight value	F7 Common factor variance	F7 Weight value
pH	0.86	0.07	0.94	0.07	0.98	0.07	0.98	0.07
NH_4^+-N	0.96	0.07	0.85	0.06	0.90	0.07	0.99	0.07
NO_3^--N	0.94	0.07	0.98	0.07	0.97	0.07	0.96	0.07
AK	0.81	0.06	0.94	0.07	0.92	0.07	0.95	0.07
AP	0.94	0.07	0.96	0.07	0.95	0.07	0.97	0.07
DOC	0.97	0.07	1.00	0.07	0.98	0.07	0.98	0.07
SOC	0.96	0.07	1.00	0.07	0.96	0.07	0.98	0.07
SOM	0.97	0.07	0.99	0.07	0.99	0.07	0.98	0.07
MBC	0.98	0.07	1.00	0.07	1.00	0.07	1.00	0.07
TN	0.96	0.07	0.99	0.07	0.99	0.07	0.99	0.07

C/N	0.95	0.07	0.99	0.07	0.96	0.07	0.97	0.07
MBN	0.97	0.07	1.00	0.07	0.99	0.07	1.00	0.07
MBC/MBN	0.98	0.08	1.00	0.07	1.00	0.07	0.99	0.07
DON	0.84	0.06	1.00	0.07	1.00	0.07	1.00	0.07

Abbreviations refer to Figure 1.

Table S4. Weights of MDS evaluation indicators of paddy soil under different treatments.

Treatments	Index	MDS	
		Common factor variance	Weight value
F3	MBC/MBN	0.98	0.26
	DOC	0.97	0.25
	NO_3^--N	0.94	0.24
	C/N	0.95	0.25
C3	MBC/MBN	1.00	0.25
	SOC	1.00	0.25
	AP	0.96	0.24
	NO_3^--N	0.98	0.25
C7	DON	1.00	0.25
	SOM	0.99	0.25
	DOC	0.98	0.25
	NO_3^--N	0.97	0.25
F7	MBN	1.00	0.25
	AP	0.97	0.25
	DOC	0.98	0.25
	pH	0.98	0.25

Abbreviations refer to Figure 1.
